# Nanotoxicity of Ultrasmall Silver Nanoclusters to Cyanobacteria

**DOI:** 10.3390/toxics14080684

**Published:** 2026-08-03

**Authors:** Xiaofei Wu, Lili Zhang, Mengqi Yin, Xiaojuan Han, Guojie Zhou, Guodong Luan, Xiaoyan Sun, Weihua Wang, Zuozhen Han, Xuefeng Lu

**Affiliations:** 1 College of Earth Science and Engineering, Shandong University of Science and Technology, Qingdao 266590, China; wuxf@qibebt.ac.cn (X.W.); hanxj@qibebt.ac.cn (X.H.); 2 State Key Laboratory of Photoelectric Conversion and Utilization of Solar Energy, Qingdao Institute of Bioenergy and Bioprocess Technology, Chinese Academy of Sciences, Qingdao 266101, China; zhangll2@qibebt.ac.cn (L.Z.); yinmq@qibebt.ac.cn (M.Y.); 15663036171@163.com (G.Z.); luangd@qibebt.ac.cn (G.L.); sunxy@qibebt.ac.cn (X.S.); lvxf@qibebt.ac.cn (X.L.); 3 Shandong Energy Institute, Qingdao 266101, China; 4 Qingdao New Energy Shandong Laboratory, Qingdao 266101, China; 5 School of Biological Science and Technology, University of Jinan, Jinan 250022, China; 6 Qingdao Marine Science and Technology Center, Qingdao 266237, China

**Keywords:** nanotoxicity, silver nanoclusters, cyanobacteria, oxidative stress, photosynthesis

## Abstract

Cyanobacteria are essential primary producers in aquatic ecosystems. Silver nanoclusters (Ag NCs) are emerging ultrasmall nanomaterials with broad applications; however, they inevitably enter aquatic environments. This study investigated the nanotoxicity of Ag NCs on model cyanobacterium *Synechococcus elongatus* PCC 7942 (Syn7942), revealing concentration-dependent growth inhibition with a 72 h half-maximal effective concentration of 1.19 mg L^−1^. Ag NCs internalized into Syn7942 cells severely impaired the photosynthetic machinery, evidenced by reduced chlorophyll a content and suppressed photochemical reactions, leading to impaired electron transport. This photosynthetic dysfunction resulted in substantial reactive oxygen species generation and lipid peroxidation, as indicated by elevated levels of malondialdehydes. Although the activities of key antioxidant enzymes were enhanced, oxidative damage ultimately overwhelmed the cellular defense capacity. Furthermore, quantitative analysis of silver ions (Ag^+^) release coupled with comparative toxicity assays of Ag^+^ and Ag NCs confirmed that the toxicity originates from the Ag NCs themselves, rather than from the released Ag^+^. Transcriptional level analysis revealed that Ag NCs altered the expression of genes involved in photosynthesis and metal detoxification. Collectively, this study reveals a unique toxicity mechanism for Ag NCs distinct from that for Ag NPs and Ag^+^, highlighting the potential ecological risks posed by ultrasmall nanomaterials.

## 1. Introduction

Cyanobacteria represent essential primary producers in aquatic environments. They contribute significantly to global primary production and play vital roles in carbon and nitrogen cycling, thereby providing the matter and energy for aquatic ecosystems [1,2,3,4]. Given their environmental prevalence and well-studied physiology, cyanobacteria are widely used as model organisms to assess the toxicity of environmental stressors, including nanoparticles [5]. Nanoparticles will eventually enter aquatic ecosystems through various pathways. The intracellular uptake of nanoparticles by cyanobacteria could facilitate the transfer, and then the accumulation of nanoparticles in higher aquatic organisms through the food chain. Therefore, nanoparticles taken up by cyanobacterial cells may affect the entire ecosystem through bioaccumulation in the food chain.

Silver nanoclusters (Ag NCs), composed of tens to hundreds of atoms and typically smaller than 3 nm, represent an emerging class of ultrasmall materials with distinctive physicochemical properties [6,7]. The ultrasmall size of Ag NCs leads to significant quantum confinement effects and a high surface-to-volume ratio, resulting in tunable fluorescence properties and excellent catalytic activity [8]. These attributes have stimulated growing interest in their potential applications across fields such as optoelectronics, catalysis, biosensing, and bioimaging [7,9,10,11,12]. However, the increasing use of Ag NCs has also raised significant concerns over their potential release into the environment and subsequent ecological impacts [13,14,15], particularly in aquatic ecosystems where nanomaterials tend to accumulate [16].

The toxic effects of silver nanoparticles (Ag NPs) and silver ions (Ag^+^) towards photosynthetic microorganisms have been extensively investigated. In terms of mechanism, the toxicity of Ag NPs in photosynthetic microorganisms is mainly mediated by the release of Ag^+^ [5,17,18,19], and these released Ag^+^ bind to thiol groups (-SH) of proteins, thereby inactivating photosynthetic enzymes [20,21,22]. This inhibition disrupts linear electron flow, leading to over-reduction of certain components in the electron transport chain and excessive production of reactive oxygen species (ROS), which ultimately causes oxidative damage [5,16,18,23].

In terms of size (<3 nm), Ag NCs represent an intermediate state between Ag^+^ and Ag NPs. Ag NCs exhibit unique semiconductor-like properties due to quantum confinement effects [8]. Ag NPs mainly serve as the release source of Ag^+^, while Ag NCs, with their extremely small size, can enter cells and directly interact with intracellular biological macromolecules [24]; simultaneously, the semiconductor-like properties might allow Ag NCs to directly interfere with photosynthetic electron transport. For photosynthetic organisms, photosynthesis is frequently a primary target of many environmental stressors. Disruption of electron flow typically leads to the excessive production of ROS, thereby triggering oxidative stress [25]. However, there is currently a lack of in-depth research regarding the mechanisms by which Ag NCs exert toxic effects on cyanobacteria.

To verify the working hypothesis regarding the toxicity of Ag NCs to cyanobacteria, this study systematically investigated the nanotoxicity of Ag NCs to the model cyanobacterium *Synechococcus elongatus* PCC 7942 (Syn7942), with an emphasis on elucidating the underlying mechanisms through multi-dimensional analysis. The findings will provide a mechanistic perspective on the cytotoxicity of Ag NCs toward cyanobacteria and facilitate a more accurate assessment of the potential ecological risks associated with this class of ultrasmall nanomaterials.

## 2. Materials and Methods

### 2.1. Synthesis and Characterization of Ag NCs

Ag NCs were synthesized according to a previously reported procedure with slight modifications [26]. In brief, silver nitrate (AgNO_3_, 0.0420 g) was dissolved in 20 mL of deionized water in an ice bath under magnetic stirring at 1000 rpm. To this solution, glutathione (GSH, 0.307 g) was added to form the silver-GSH complex. The pH was then adjusted to 7 by the dropwise addition of a NaOH solution, resulting in the complete dissolution of the white precipitate and a clear mixture. Subsequently, 2 mL of an ice-cold aqueous solution of NaBH_4_ (0.0950 g, 2.50 mmol) was added slowly under vigorous stirring. The reaction mixture was then allowed to proceed and aged at room temperature for 7 h. To purify the Ag NCs, the reaction solution was mixed with 150 mL of analytical-grade methanol (≥99.9%) added in a single portion to precipitate the crude product. The precipitate was collected by centrifugation, redissolved in 2 mL of water, and centrifuged again to remove insoluble impurities. The supernatant, containing the water-soluble nanoclusters, was collected and mixed with 150 mL of methanol to reprecipitate the purified nanoclusters. This redissolution-precipitation cycle was repeated three times to obtain the final purified Ag NCs.

Morphological and size characteristics of the Ag NCs were determined using transmission electron microscopy (TEM, JEM-F200, JEOL Ltd., Tokyo, Japan). Molecular weight was determined by high-resolution electrospray ionization mass spectrometry (ESI-MS) using a Waters time-of-flight mass spectrometer (Waters Corporation, Milford, MA, USA), operating in negative ion mode with a mass scan range of *m/z* = 50–2000. For the analysis, the sample was dissolved and diluted to approximately 10.0 mg L^−1^ in deionized water. Measurement parameters were as follows: capillary voltage, 2.50 kV; sampling cone, 40.0; source offset, 80.0; source temperature, 100 °C; desolvation temperature, 250 °C; cone hole flow, 50.0 L h^−1^; desolvation gas flow, 600 L h^−1^; and nebulizer gas pressure, 6.50 bar.

### 2.2. Cultivation of Syn7942 with Ag NCs

The Syn7942 strain used in this study was obtained from Prof. Xudong Xu’s lab at the Institute of Hydrobiology, Chinese Academy of Sciences. The growth of Syn7942 was monitored in the presence of varying concentrations of Ag NCs. Cells were inoculated into BG11 medium at an initial optical density at 730 nm (OD_730_) of 0.1. The treatment groups were added with Ag NCs to achieve final concentrations of 0, 0.05, 0.5, 1, 3, and 5 mg L^−1^, respectively. Each culture was grown in a glass column (3 cm in diameter and 20 cm high) at 30 °C under continuous white-light illumination (100 μmol photons m^−2^ s^−1^), and aerated with 3% CO_2_. Growth was tracked over 72 h by measuring OD_730_ and cell density (measured using an IA1000 Countstar BioMarine cell counter, Shanghai, China [27]) every 24 h. Control measurements of Ag NCs in cell-free BG11 medium confirmed that their contribution to OD_730_ was negligible.

### 2.3. Determination of Chlorophyll a Content

The chlorophyll *a* (Chl *a*) content was determined using a modified methanol extraction method [28]. Specifically, 2 mL of cell culture was centrifuged, and the pellet was resuspended in 2 mL of methanol. The mixture was incubated in the dark at −20 °C for 16 h. Subsequently, the sample was centrifuged at 10,000× *g* for 5 min at 4 °C. The absorbance of the supernatant was measured at 665 nm and 720 nm using a spectrophotometer (Yuanxi UV-5100, Shanghai, China). The Chl *a* content was calculated using the following equation:

Chl *a* (μg mL^−1^) = 12.9447 × (A_665_ − A_720_)

### 2.4. Determination of the Photosynthetic O_2_ Evolution Rate

The photosynthetic O_2_ evolution rate of Syn7942 and Ag NCs-treated (Ag-Syn7942) cells was determined using a YZQ-201A photosynthetic instrument (Beijing Yaxinliyi Technology, Beijing, China). After 72 h of exposure to Ag NCs at the half-maximal effective concentration (EC_50_), Syn7942 and Ag-Syn7942 cells were harvested and resuspended in fresh BG11 medium to an OD_730_ of 1.00. All measurements were carried out at 30 °C in the presence of 10 mM NaHCO_3_ as an inorganic carbon source. The respiratory O_2_ consumption rate was measured in the dark, and the net photosynthetic O_2_ evolution rate was measured under various light intensities (RGB light source). The total photosynthetic O_2_ evolution rate was calculated as the sum of the net photosynthetic rate and the absolute value of the respiration rate.

### 2.5. Determination of the Chlorophyll Fluorescence and P700

After 72 h of exposure to Ag NCs at the EC_50_ concentration, Syn7942 and Ag-Syn7942 cells were harvested and resuspended to a density of approximately 4 × 10^8^ cells in 2 mL. Both light curves and induction curves of chlorophyll fluorescence and P700 were measured using the Dual-PAM-100 fluorometer (Heinz Walz GmbH, Effeltrich, Germany). Before induction curve measurements, the samples were dark-acclimated for 20 min at 30 °C. The recorded parameters included minimum fluorescence (F_0_), steady-state fluorescence (F), maximum fluorescence under light adaptation (F_m_′), maximum P700 oxidation level (P_m_), maximum P700 oxidation level under light adaptation (P_m_′), and P700 oxidation level (P700_ox_). Maximum fluorescence (F_m_) was determined following the addition of 20 μM 3-(3,4-dichlorophenyl)-1,1-dimethylurea (DCMU) under actinic light. The maximum quantum yield (F_v_/F_m_), actual photosynthetic efficiency of PSII [Y(II)] and actual photosynthetic efficiency of PSI [Y(I)] were calculated according to the equations in Table S1.

### 2.6. Determination of Intracellular ROS Levels

Intracellular ROS levels in Syn7942 and Ag-Syn7942 cells were measured using the fluorescent probe 2′,7′-dichlorofluorescein diacetate (DCFH-DA) from a commercial Reactive Oxygen Species Assay Kit (Beyotime, Shanghai, China). After 72 h of exposure to Ag NCs at the EC_50_ concentration, approximately 1 × 10^8^ cells were collected and resuspended in 1 mL of fresh BG11 medium supplemented with 10 μM DCFH-DA, and incubated at 37 °C for 20 min with gentle shaking. After incubation, the cells were washed three times with BG11 to remove extracellular dye. Fluorescence intensity was recorded with a SpectraMax M3 microplate reader (Molecular Devices, San Jose, CA, USA) at excitation and emission wavelengths of 488 nm and 525 nm, respectively.

### 2.7. Determination of Antioxidant Enzyme Activities and Malondialdehyde Content

Following 72 h exposure to Ag NCs at the EC_50_ concentration, Syn7942 and Ag-Syn7942 cells were harvested by centrifugation at 6000× *g* for 10 min. Cell pellets were resuspended in 500 μL of extraction buffer and disrupted by ultrasonication on ice. The lysates were centrifuged at 8000× *g* for 10 min at 4 °C, and the supernatants were collected and analyzed according to the corresponding commercial assay kits.

The superoxide dismutase (SOD) activity was measured using a WST-based assay kit (BC5165, Solarbio, Beijing, China). The catalase (CAT) activity was determined using a catalase activity detection kit (BC0205, Solarbio), and the peroxidase (POD) activity was assessed with a peroxidase activity assay kit (BC0095, Solarbio). Malondialdehyde (MDA), a marker of lipid peroxidation, was measured using a Malondialdehyde content detection kit (BC0025, Solarbio).

To normalize for variations in protein content during sample preparation, the total protein concentration in the supernatants was determined using the Bradford method with bovine serum albumin (BSA) as the standard. Antioxidant enzyme activities (SOD, CAT, POD) were expressed as units per milligram of protein (U mg^−1^ protein), while the MDA content was given in nanomoles per milligram of protein (nmol mg^−1^ protein). All absorbance measurements were recorded using a SpectraMax M3 microplate reader (Molecular Devices).

### 2.8. Quantification of Silver Ion Release

The release of Ag^+^ from Ag NCs was quantified using inductively coupled plasma mass spectrometry (ICP-MS, ICP-MS-2030, Shimadzu, Japan) [29]. BG11 medium containing 4.00 mg L^−1^ Ag NCs was incubated in a glass column (20 cm high and 3 cm in diameter) under continuous white-light illumination (100 μmol photons m^−2^ s^−1^) and aerated with 3% CO_2_ at 30 °C. After 72 h of incubation, culture samples were collected and sequentially filtered through a 0.1 μm filter and an ultrafiltration tube with a molecular weight cutoff of 3 kDa (Merck Millipore, Darmstadt, Germany). The concentration of released Ag ions from Ag NCs was further analyzed by ICP-MS. The total silver content in the initial 4 mg L^−1^ Ag NCs was also determined by ICP-MS.

### 2.9. Scanning Transmission Electron Microscopy

Syn7942 cells and Ag-Syn7942 were harvested by centrifugation at 4000 rpm for 5 min. The pellets were washed twice with 0.9% NaCl buffer for 15 min each. The cells were then fixed in 2.5% glutaraldehyde at 4 °C for 24 h. After fixation, the samples were washed three times with 0.9% NaCl buffer (20 min each) with gentle resuspension. Dehydration was carried out using a graded acetone series (30%, 50%, 70%, 80%, 90%, 95%) with each step lasting 15 min, followed by three 15-min treatments in 100% acetone. The samples were then infiltrated with resin: first with a 7:3 (*v*/*v*) acetone: resin mixture for 5 h, followed by a 3:7 mixture for another 5 h, and finally with pure resin for 12 h. After infiltration, the samples were embedded in resin molds and polymerized at 70 °C for 24 h. Ultrathin sections (approximately 70 nm) were prepared and observed using the scanning transmission electron microscopy (STEM) mode of a high-resolution transmission electron microscope (JEM-F200).

### 2.10. Cysteine-Silver Complexation Experiments

To differentiate the toxic effects of Ag NCs from those of released Ag^+^, a complexation experiment was conducted using L-cysteine (L-Cys). L-Cys, a thiol-containing amino acid, can specifically chelate free Ag^+^, thereby mitigating its biological toxicity.

Syn7942 cells were inoculated into BG11 medium at an initial OD_730_ of 0.1. Four treatment groups were established: (i) BG11 medium (control); (ii) BG11 medium supplemented with 0.2 μg L^−1^ Ag^+^, corresponding to the concentration of Ag^+^ released from 4 mg L^−1^ Ag NCs; (iii) BG11 medium supplemented with 55 μg L^−1^ Ag^+^ representing the total silver content equivalent to 4 mg L^−1^ Ag NCs; (iv) BG11 medium supplemented with 4 mg L^−1^ Ag NCs. For each of the above 4 treatments, both the absence and the presence of L-Cys were covered. The concentration of L-Cys was 0.0255 mM (50:1 molar ratio relative to 55 μg L^−1^ Ag^+^). Each culture was grown in a glass column (3 cm in diameter and 20 cm high) at 30 °C under continuous white-light illumination (100 μmol photons m^−2^ s^−1^), and aerated with 3% CO_2_. After 72 h of cultivation, OD_730_, cell density, and Chl *a* content were measured. In addition, the whole-chain photosynthetic oxygen evolution rate and F_v_/F_m_ were also determined.

### 2.11. RNA Extraction and RT-qPCR Analysis

To investigate the molecular responses of Syn7942 to Ag NCs and Ag^+^, reverse transcription quantitative real-time PCR (RT-qPCR) was performed. Syn7942 cells were exposed to 4 mg L^−1^ Ag NCs or 55 μg L^−1^ Ag^+^ for 72 h. After treatment, cells were harvested by centrifugation at 6000× *g* for 10 min at 4 °C. Total RNA was extracted using RNA Isolator Total RNA Extraction Reagent (Vazyme, Nanjing, China). RNA concentration and purity were determined using a NanoDrop 2000 spectrophotometer (Thermo Scientific, Waltham, MA, USA). Genomic DNA removal and reverse transcription were performed using the HiScript III RT SuperMix for qPCR (+gDNA wiper) (Vazyme, China). RT-qPCR was performed using ChamQ Universal SYBR qPCR Master Mix (Vazyme, China) on a LightCycler480 Real-Time PCR Detection System (Roche Applied Science, Basel, Switzerland). The *RecA* gene was used as the internal control. The relative expression levels of target genes (listed in Table S2) were calculated using the 2^−ΔΔCt^ method.

### 2.12. Statistical Analysis

All experiments were performed with four independent biological replicates, and data are presented as mean ± standard deviation (SD). Statistical analyses were performed using GraphPad Prism version 9.0 (GraphPad Software, San Diego, CA, USA). Normality and homogeneity of variance were checked before analysis. A two-tailed unpaired Student’s *t*-test was used for two-group comparisons, while one-way ANOVA with Dunnett’s test was applied for multi-group comparisons against the control. The EC_50_ value was calculated by fitting the concentration-response data to a logistic nonlinear regression model using GraphPad Prism 9.0. A *p*-value < 0.05 was considered statistically significant. Significance levels are indicated as follows: * *p* < 0.05, ** *p* < 0.01, and *** *p* < 0.001.

## 3. Results

### 3.1. Characterization of Ag NCs

**Figure 1:**
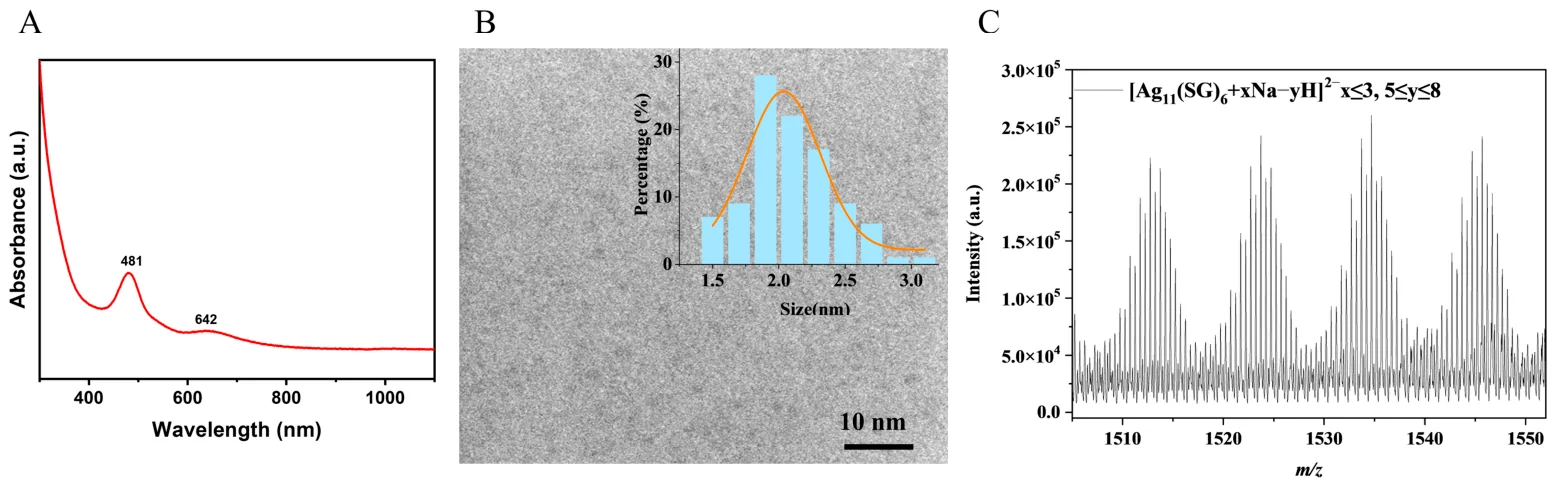
The characterization of GSH-protected Ag NCs. (**A**). UV-vis absorption spectrum of Ag NCs. (**B**). TEM image of Ag NCs at a scale of 10 nm, with the inset showing the size distribution histogram. (**C**). Negative-ion mode ESI-MS spectrum of Ag NCs.

The GSH-protected Ag NCs were characterized through a combination of ultraviolet-visible (UV-vis) absorption spectroscopy, TEM images, and high-resolution ESI-MS analysis. As shown in the UV-vis absorption spectrum (Figure 1A), the Ag NCs exhibited two distinct absorption peaks at 481 nm and 642 nm, which are consistent with those for reported GSH-protected Ag NCs [30,31], indicating good monodispersity. TEM analysis was conducted to investigate the morphology and size distribution of the Ag NCs. As shown in Figure 1B, TEM images demonstrated that the sample predominantly consisted of well-dispersed, ultrasmall nanoparticles with a uniform shape. Statistical analysis of over 100 individual particles revealed a narrow size distribution, with an average diameter of 2.10 ± 0.30 nm, as shown in the inset histogram of Figure 1B, consistent with the typical dimensions of nanoclusters.

To further determine the composition of the Ag NCs, ESI-MS analysis was performed in the negative ion mode. As shown in Figure 1C, a series of Ag NC peaks were observed at around *m/z* = 1500, which can be assigned to [Ag_11_(SG)_6_ + xNa − yH]^2−^ (where x ≤ 3 and 5 ≤ y ≤ 8) (see Figure S1 for full isotope matching details). Among them, the peak at *m/z* = 1512 corresponds to [Ag_11_(SG)_6_−5H]^2−^. The excellent agreement between the experimental and theoretical *m/z* values strongly confirms the presence of this species. Overall, the good dispersibility and well-defined composition of the Ag NCs were confirmed by the above characterizations.

### 3.2. Cellular Uptake and Growth Inhibition of Ag NCs in Syn7942

**Figure 2:**
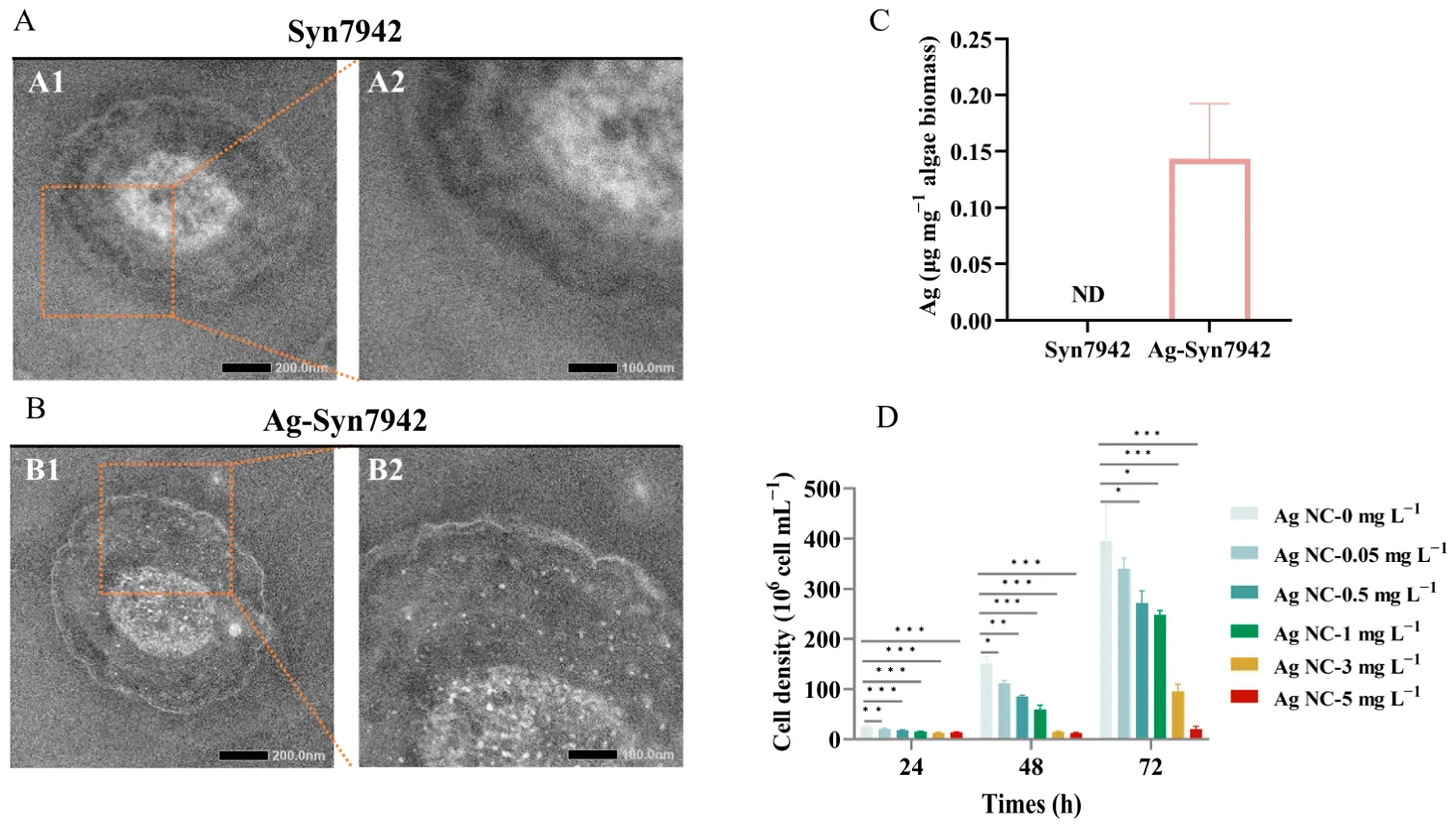
Cellular uptake and growth inhibition of Ag NCs in Syn7942. (**A**). STEM images of Syn7942 cells at 200 nm (**A1**) and 100 nm (**A2**) scales. (**B**). STEM images of Ag-Syn7942 cells at 200 nm (**B1**) and 100 nm (**B2**) scales; The distinct white spots correspond to Ag NCs within Syn7942 cells. (**C**). Quantitative analysis of intracellular silver in Syn7942 cells by ICP-MS. (**D**). Cell density of Syn7942 cells exposed to varying concentrations of Ag NCs. Data are presented as Mean ± SD. * *p* < 0.05, ** *p* < 0.01, and *** *p* < 0.001. ND indicates not detected (below the detection limit).

To investigate the cellular localization of Ag NCs in cyanobacteria, STEM imaging was conducted on both Syn7942 and Ag NCs-treated Syn7942 (Ag-Syn7942) cells. In dark field mode, materials with high atomic numbers, such as silver, scatter electrons strongly and appear as bright features against the darker cellular background. As shown in Figure 2A, STEM images of Syn7942 cells revealed a homogeneous, low-contrast cytoplasm at both 200 nm (A1) and 100 nm (A2) magnifications, with no bright nanoscale particles. In contrast, Syn7942 cells exposed to Ag NCs (Figure 2B) exhibited numerous bright, nanoscale dots scattered throughout the intracellular space at corresponding magnifications (B1, B2). These distinct bright spots, which were absent in control cells, provided visual evidence for the intracellular localization of Ag NCs, confirming the internalization of Ag NCs by Syn7942. ICP-MS analysis (Figure 2C) further confirmed this observation, as no detectable Ag was found in control cell samples, while a distinct Ag signal was detected in the Ag-Syn7942 cell samples. It is indicated that the ultrasmall size and excellent dispersibility of the Ag NCs enabled them to be internalized by Syn7942 cells. The intracellular distribution of Ag NCs could facilitate their interaction with cellular components, which may influence the growth of Syn7942 cells.

The effect of Ag NCs on the growth of Syn7942 was assessed by inoculating the cyanobacterium into BG11 medium supplemented with varying concentrations of Ag NCs (0, 0.05, 0.5, 1, 3, and 5 mg L^−1^). Growth was assessed by monitoring the OD_730_ value over a period of 72 h. (Figure S2). Even at the lowest concentration (0.05 mg L^−1^), Ag NCs induced a significant suppression of growth, while nearly complete inhibition was observed at 5 mg L^−1^. These results were confirmed by cell density measurements, which showed a concentration-dependent reduction in viable cell counts (Figure 2D). The content of Chl *a*, an essential photosynthetic pigment responsible for light harvesting and excitation energy transfer within the photosynthetic apparatus, was also quantified. As shown in Figure S3, Chl *a* content exhibited a significant concentration-dependent reduction upon exposure to Ag NCs. This reduction suggested an impairment in photosynthesis and possible interference with chlorophyll biosynthesis, which likely caused the observed growth inhibition.

After 72 h of treatment, the growth inhibition rates relative to the Ag NC-free control were determined as 13.9% (0.05 mg L^−1^), 28.1% (0.5 mg L^−1^), 39.3% (1 mg L^−1^), 75.8% (3 mg L^−1^), and 94.8% (5 mg L^−1^). A concentration-dependent relationship was established using logistic nonlinear regression (*R^2^* = 0.920), from which the EC_50_ was estimated to be 1.19 mg L^−1^ (95% confidence interval: 0.970–1.43 mg L^−1^) (Figure S4).

### 3.3. Ag NCs Impair Photosynthesis Activity and Induce Oxidative Stress in Syn7942

**Figure 3:**
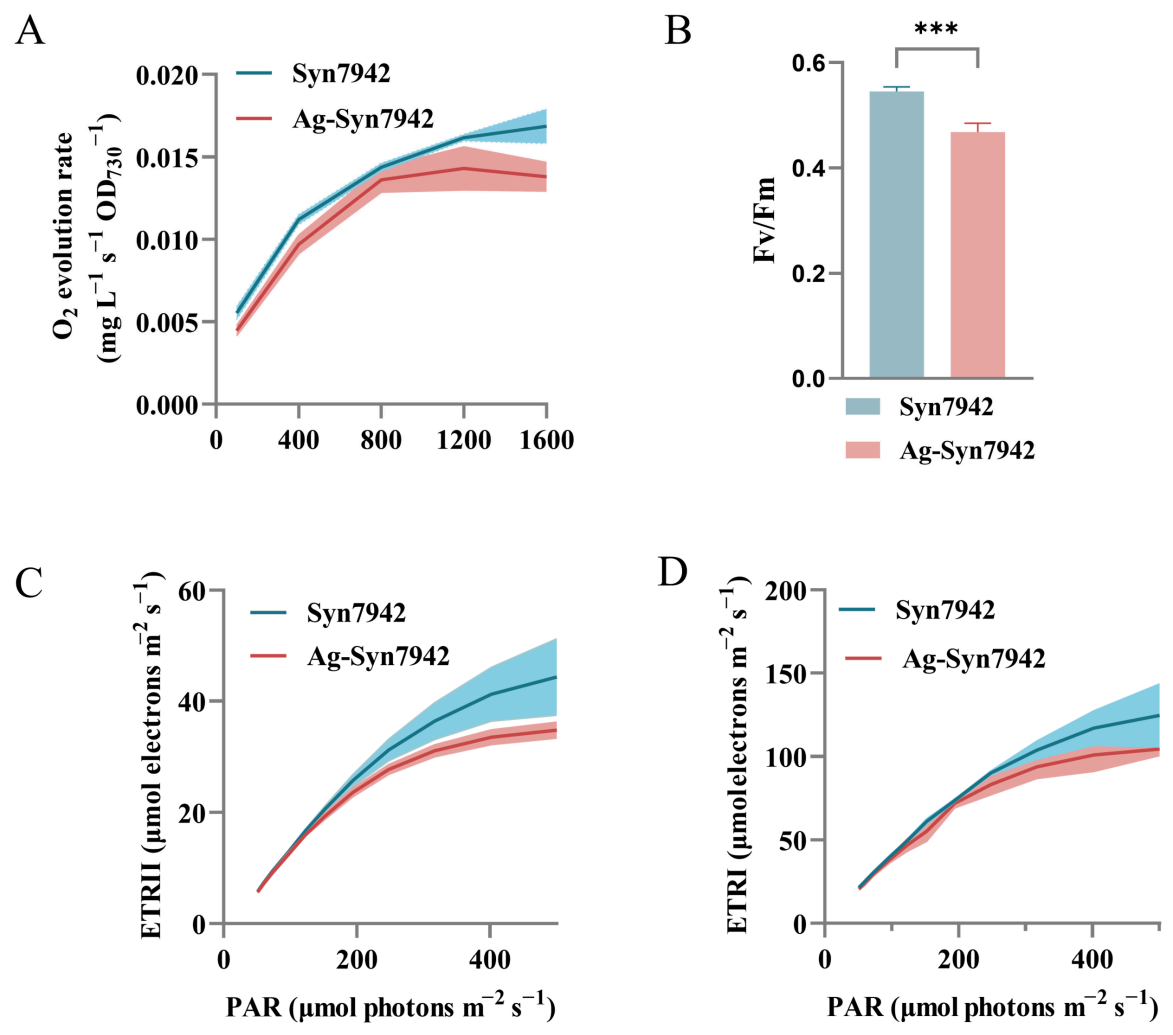
Effects of Ag NCs on photosynthetic activity in Syn7942 after 72 h exposure at the EC_50_ concentration. (**A**). Whole-chain photosynthetic oxygen evolution rate under varying light intensities. (**B**). Maximum quantum efficiency of PS II (F_v_/F_m_). (**C**). Electron transport rate of PSII [ETR(II)]. (**D**). Electron transport rate of PSI [ETR(I)]. Data are presented as Mean ± SD. *** *p* < 0.001.

The observed growth inhibition of Syn7492 and the concomitant concentration-dependent decrease in Chl *a* content upon exposure to Ag NCs suggest potential impairment of photosynthesis. So, we analyzed key photosynthetic parameters after 72 h of exposure to Ag NCs at the EC_50_. Exposure to Ag NCs significantly inhibited major photosynthetic processes in Syn7942. The dark respiratory oxygen consumption rate decreased from 0.00161 mg L^−1^ s^−1^ OD_730_^−1^ to 0.00103 mg L^−1^ s^−1^ OD_730_^−1^ (Figure S5), indicating compromised respiratory activity. Concurrently, the whole-chain photosynthetic oxygen evolution rate decreased at varying light intensities (Figure 3A), demonstrating a direct inhibition of overall photosynthetic capacity.

The maximum photochemical efficiency of PSII, represented by the F_v_/F_m_ ratio, decreased by 14.1% from 0.545 to 0.468 (Figure 3B), indicating damage to the PSII reaction centers and a reduction in the potential efficiency of photochemical conversion. This was consistent with the decrease in the effective quantum yield of PSII [Y(II)] from 0.316 to 0.290 (Figure S6), a decline of 8.2%, which reflected a lower actual efficiency of light energy conversion under steady-state illumination. The impairment extended to the photosynthetic electron transport chain, as evidenced by the significant decrease in the electron transport rate of PSII [ETR(II)] (Figure 3C). The activity of PSI was also inhibited with its quantum yield [Y(I)] declining by 6.5% from 0.949 to 0.887 (Figure S6). This reduction in PSI activity was likely an indirect effect resulting from the primary damage to PSII. The impaired electron flow from PSII reduced the electron supply to PSI, thereby limiting its photochemical activity. This interpretation was further supported by the concomitant decrease in the electron transport rate of PSI [ETR(I)] (Figure 3D).

**Figure 4:**
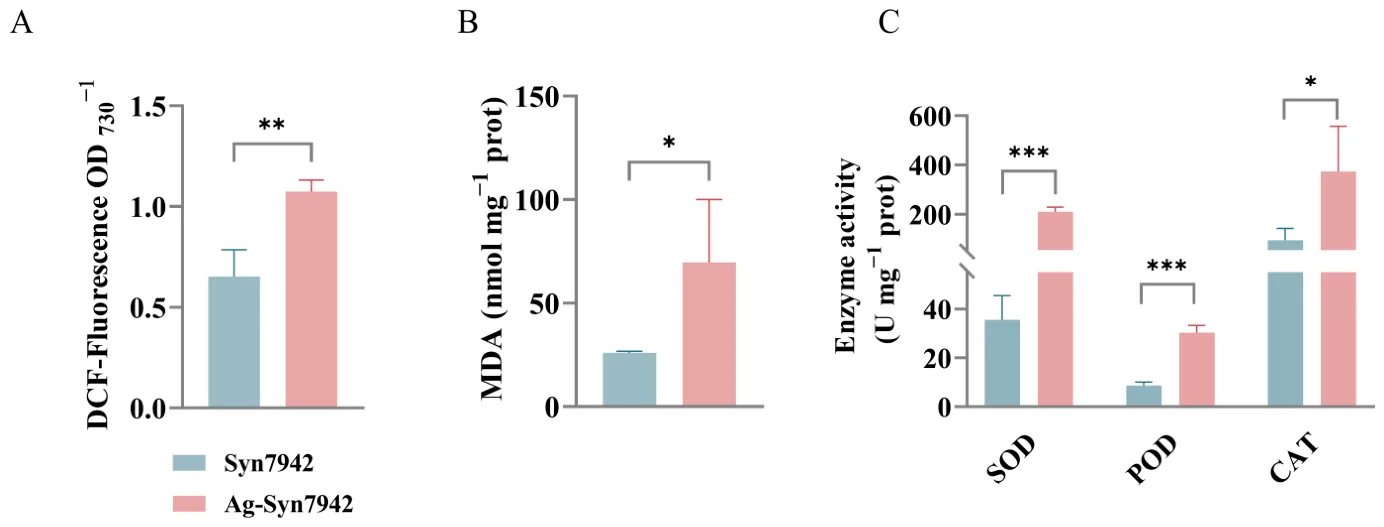
Effects of Ag NCs on oxidative stress in Syn7942 after 72 h exposure at the EC_50_ concentration. (**A**). Intracellular ROS levels. (**B**). MDA content as a marker of lipid peroxidation. (**C**). SOD activity, POD activity and CAT activity. Data are presented as Mean ± SD. * *p* < 0.05, ** *p* < 0.01, and *** *p* < 0.001.

The impairment of the photosynthetic electron transport chain by Ag NCs would induce an over-reduction within the photosynthetic systems, promoting ROS generation and triggering oxidative stress. Indeed, after 72 h of cultivation with Ag NCs at the EC_50_, intracellular ROS levels were significantly increased by 63.9%, from 0.653 to 1.07 (Figure 4A). This accumulation was a direct consequence of the blockage of the photosynthetic electron transport chain, in which case excess electrons are transferred to O_2_, generating ROS. A significant increase in lipid peroxidation further evidenced the ensuing oxidative damage. Elevated ROS levels would lead to a significant rise in MDA content, a well-established biomarker of membrane damage, from 25.9 to 69.6 nmol mg^−1^ protein (Figure 4B), an increase of 168.7%. This result provided direct evidence that excessive production of ROS leads to severe damage to cell membranes.

In response to oxidative stress, Syn7942 activated a coordinated antioxidant system. The activities of key antioxidant enzymes were significantly elevated. SOD activity, which plays a key role in scavenging superoxide radicals, increased from 35.6 U mg^−1^ protein to 210 U mg^−1^ protein (489.9% increase) (Figure 4C). Similarly, A concurrent increase in POD activity, which facilitates the detoxification of peroxides, was observed, rising from 8.69 U mg^−1^ protein to 30.3 U mg^−1^ protein (248.7% increase). CAT activity, which is responsible for the decomposition of hydrogen peroxide, increased from 95.8 U mg^−1^ protein to 373 U mg^−1^ protein (289.4% increase). The coordinated upregulation of SOD, POD, and CAT activities indicated that Syn7942 established an instant antioxidant defense to mitigate ROS-induced cell damage. However, the sustained elevation of ROS levels and the pronounced lipid peroxidation demonstrated that this defense was ultimately overwhelmed. The inability to fully counteract the oxidative stress is a major factor contributing to growth inhibition.

### 3.4. Differentiating the Toxic Contributions of Ag NCs and Released Ag^+^

ICP-MS analysis was conducted to quantify the Ag^+^ release of the Ag NCs in the culture medium. As shown in Figure S7A, the concentration of Ag^+^ released from 4 mg L^−1^ Ag NCs after 72 h was 0.2 μg L^−1^. The total silver content in the 4 mg L^−1^ Ag NCs was 55 μg L^−1^ (Figure S7B). It is indicated that the released Ag^+^ accounted for only a minor fraction (~ 0.36%) of the total silver content in the Ag NCs with the majority of silver existing in nanocluster form. Due to the presence of protecting ligands, the Ag NCs exhibit good stability, and only a very small amount of Ag^+^ was released into the culture medium.

**Figure 5:**
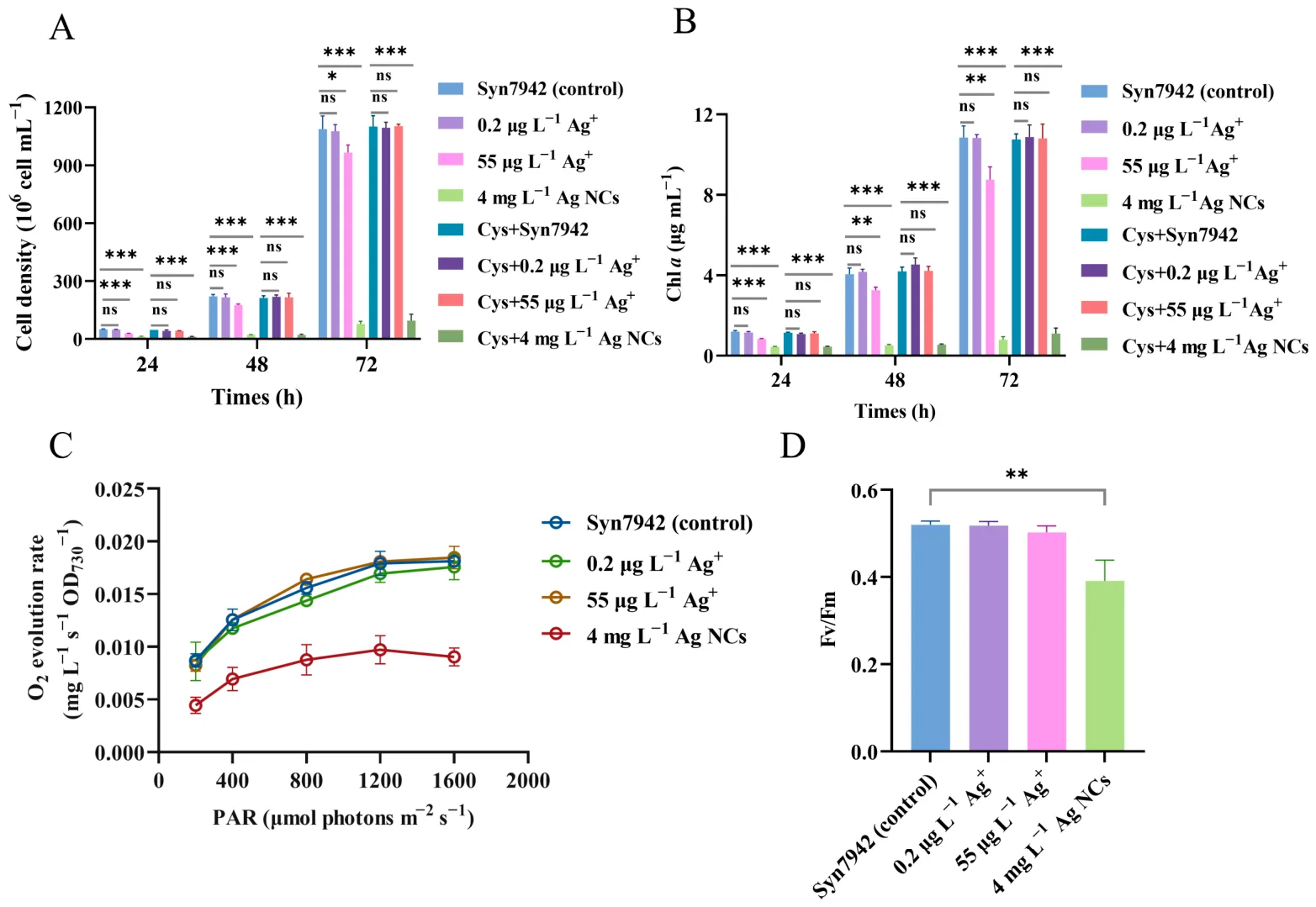
Comparative assessment of the effects of Ag NCs and Ag^+^ on growth and photosynthesis of Syn7942. (**A**). Cell density of Syn7942 under different culture conditions: BG-11 medium, BG-11 medium containing 0.2 μg L^−1^ Ag^+^ with or without L-Cys, BG-11 medium containing 55 μg L^−1^ Ag^+^ with or without L-Cys, and BG-11 medium containing 4 mg L^−1^ Ag NCs with or without L-Cys. (**B**). Chl *a* content of cells after 72 h exposure under different culture conditions. (**C**). The whole-chain photosynthetic oxygen evolution rate under varying light intensities. (**D**). F_v_/F_m_. Data are presented as Mean ± SD. * *p* < 0.05, ** *p* < 0.01, and *** *p* < 0.001. ns indicates not significant.

To directly compare the toxic effects of Ag^+^ and Ag NCs, Syn7942 was cultured under 4 conditions: (i) BG-11 medium (control), (ii) BG-11 medium containing 0.2 μg L^−1^ Ag^+^, (iii) BG-11 medium containing 55 μg L^−1^ Ag^+^, and (iv) BG-11 medium containing 4 mg L^−1^ Ag NCs. Comparison of the growth curves (Figure S8), cell density (Figure 5A) and Chl *a* content (Figure 5B) revealed that the 0.2 μg L^−1^ Ag^+^ addition did not significantly inhibit growth of Syn7942. Exposure of Syn7942 to 55 μg L^−1^ Ag^+^ induced slight growth inhibition, with an inhibition rate of 11.22% at 72 h. However, the growth inhibition of Syn7942 by 4 mg L^−1^ Ag NCs was much more significant, reaching 92.88% at 72 h. To further validate the source of toxicity, an L-Cys complexation experiment was performed. L-Cys acts as a strong thiol ligand that specifically chelates free Ag^+^, effectively neutralizing its cytotoxicity [19]. The addition of L-Cys (0.0255 mM) completely abolished the growth inhibition by 55 μg L^−1^ Ag^+^, allowing Syn7942 to recover to control-like levels. Following the addition of L-Cys, the inhibitory effect of 4 mg L^−1^ Ag NCs persisted without significant change. Furthermore, while 0.2 μg L^−1^ and 55 μg L^−1^ Ag^+^ caused no significant decline in the whole-chain photosynthetic oxygen evolution rate (Figure 5C) and F_v_/F_m_ (Figure 5D), Ag NCs significantly reduced the photosynthetic oxygen evolution rate and F_v_/F_m_ decreased by 24.8%, confirming damage to the PSII reaction center. These results indicate that the nanotoxicity of Ag NCs stems primarily from their semiconductor-like properties, rather than from the released Ag^+^.

### 3.5. Molecular Mechanisms of Ag NC Toxicity Revealed by Targeted Gene Expression Analysis

**Figure 6:**
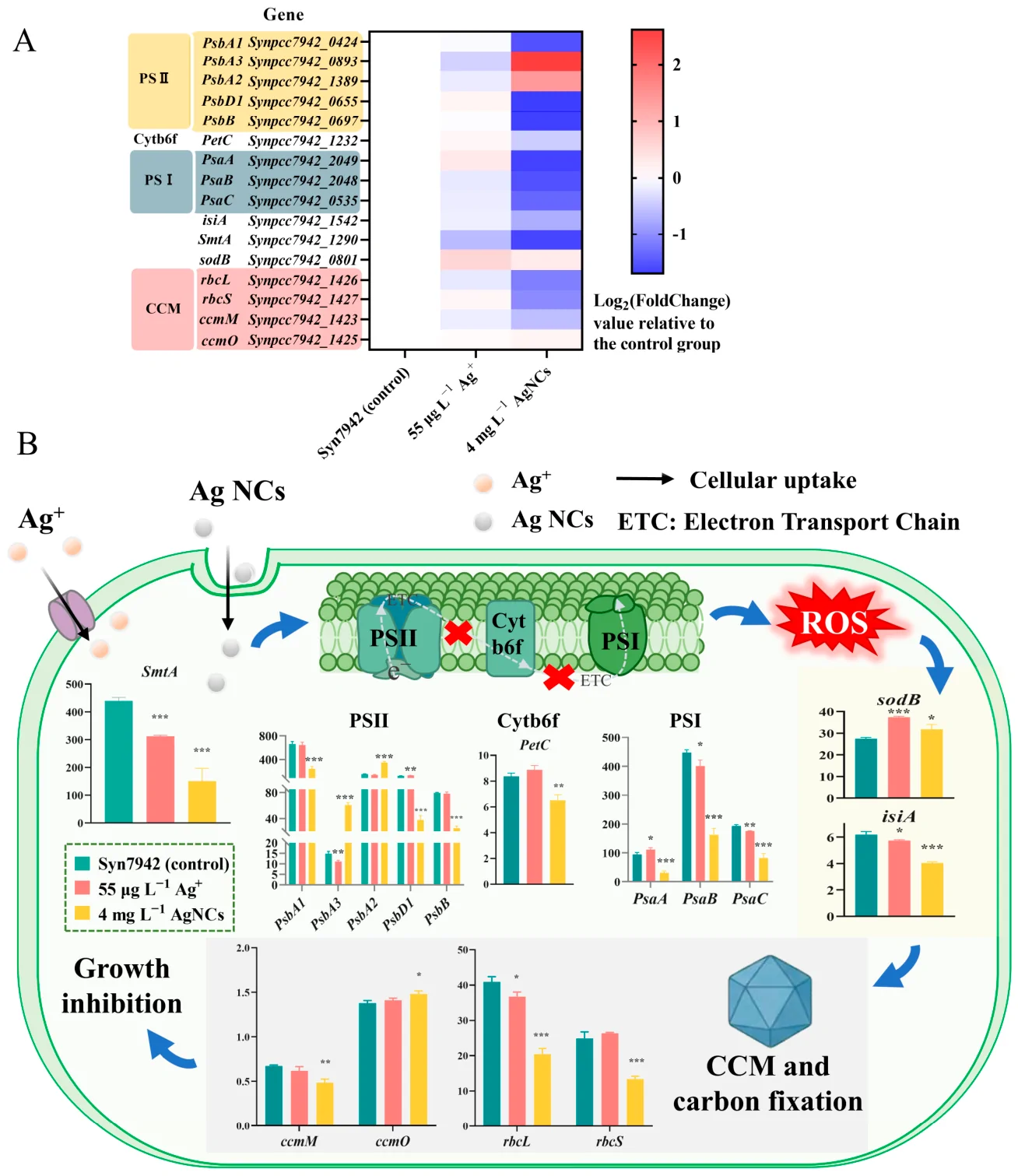
Molecular mechanisms of Ag NC and Ag^+^ toxicity to Syn7942 revealed by RT-qPCR analysis. (**A**). Heatmap showing log_2_(Fold Change) of 16 target genes involved in PSII, cytochrome b6f (Cytb6f), PSI, metallothionein (SmtA), antioxidant defense (sodB), and the carbon-concentrating mechanism (CCM) following exposure to Ag^+^ and Ag NCs. Blue indicates downregulation and red indicates upregulation relative to the control. (**B**). Schematic model of the intracellular toxic pathways. The bar charts illustrate in detail the relative expression levels of key target genes in each treatment group, with the *y*-axis representing relative expression levels (2^−ΔCt^). The gray dashed line indicates the electron transport chain (ETC; from PSII through Cytb6f to PSI). Red crosses indicate inhibition or disruption of the ETC. Black arrows indicate internalization (cellular uptake). Data are presented as Mean ± SD. * *p* < 0.05, ** *p* < 0.01, and *** *p* < 0.001.

To elucidate the molecular mechanisms of Ag NC toxicity, RT-qPCR analysis was conducted on 16 target genes associated with photosynthesis, antioxidant defense, metal sequestration, and carbon fixation. Gene expression profiles of Syn7942 cells exposed to Ag NCs (4 mg L^−1^) were compared with those treated with an equivalent total silver content of Ag^+^ (55 μg L^−1^). The changes in gene expression relative to the control group were presented in Figure 6A, while the detailed transcriptional changes in individual genes are shown in Figure 6B.

The expression profiles revealed profound differences between Ag NCs and Ag^+^ treatments. Compared to Ag^+^, exposure to Ag NCs resulted in a more significant downregulation of *psbA1* (*Synpcc7942_0424*), accompanied by the substantial upregulation of *psbA2* (*Synpcc7942_1389*) and *psbA3* (*Synpcc7942_0893*). Additionally, Ag NCs downregulated *psbD1* (*Synpcc7942_0655*), encoding the D2 protein, and *psbB* (*Synpcc7942_0697*), encoding CP47, whereas Ag^+^ induced a compensatory upregulation of *psbD1*. Beyond PSII, Ag NCs caused a widespread suppression of genes encoding key components of the cytochrome b6f complex (*petC*, *Synpcc7942_1232*) and PSI subunits (*psaA*, *psaB*, and *psaC*). In contrast, Ag^+^ induced the upregulation of *psaA* and *petC*.

The photoprotection-related gene *isiA* (*Synpcc7942_1542*) was significantly more downregulated under Ag NC exposure than under Ag^+^ treatment. The antioxidant defense gene *sodB* (*Synpcc7942_0801*) was significantly upregulated following Ag NC treatment. Compared with Ag^+^, the metallothionein gene *smtA* (*Synpcc7942_1290*) exhibited a pronounced downregulation under Ag NC exposure.

Genes associated with carbon fixation were also affected. Both RuBisCO subunits (*rbcL*, *Synpcc7942_1426*; *rbcS*, *Synpcc7942_1427*) were significantly downregulated by Ag NCs, whereas Ag^+^ only affected *rbcL*. Within the carbon-concentrating mechanism, Ag NCs suppressed the carboxysome core protein (*ccmM*, *Synpcc7942_1423*) but upregulated the shell protein-encoding gene (*ccmO*, *Synpcc7942_1425*).

Overall, Ag NCs induced broader and more pronounced transcriptional perturbations than equivalent concentrations of Ag^+^.

## 4. Discussion

This study systematically investigated the toxic effects of ultrasmall Ag NCs on the model cyanobacterium Syn7942 (manifesting as impaired photosynthesis, oxidative stress, and consequent growth inhibition) as well as the underlying molecular mechanisms. The Ag NCs possessed an ultrasmall particle size (2.10 ± 0.30 nm), excellent dispersibility, and a stable cluster structure, properties that facilitated their internalization into the cyanobacterial cells (Figure 1). The accumulation of Ag NCs within Syn7942 cells was confirmed through STEM imaging and ICP-MS analysis (Figure 2A). Such ultrasmall dimensions likely promote not only cellular uptake but also intracellular trafficking and sustained biointeractions with subcellular targets [32]. This phenomenon is similarly observed in cyanobacteria exposed to other small-sized nanomaterials, such as gold nanoclusters and carbon quantum dots [33,34]. As a consequence of internalization, Ag NCs induced a concentration-dependent growth inhibition effect on Syn7942, with a 72 h EC_50_ of 1.19 mg L^−1^, indicating a high sensitivity of the cyanobacteria to the Ag NCs (Figure 2C,D). Compared to eukaryotic microalgae, Syn7942 exhibited a higher sensitivity to Ag NCs [24]; this may be attributed to the absence of membrane-bound organelles (such as chloroplasts) in cyanobacteria, which renders their intracellular macromolecules more susceptible to direct damage.

The physiological analysis revealed that photosystems are a primary target of Ag NCs toxicity. Ag NCs exposure significantly reduced Chl *a* content, photosynthetic oxygen evolution, and the electron transport rates of both PSII and PSI, indicating severe impairment of the photosynthetic electron transport chain (Figure 3). Since the photosynthetic complexes in cyanobacteria are directly embedded within the thylakoid membranes, the internalized Ag NCs could directly interfere with the electron transfer process. This interference likely leads to the over-reduction of electron carriers and promotes the excessive generation of ROS. The results revealed that following exposure to Ag NCs, intracellular ROS and MDA levels increased significantly, indicating that the cells had sustained severe oxidative stress and membrane lipid peroxidation (Figure 4). Although Syn7942 activated antioxidant defenses by enhancing the activities of SOD, POD, and CAT, these responses were insufficient to completely scavenge the accumulated ROS, suggesting that oxidative damage had exceeded the cellular detoxification capacity [29,35,36].

An important finding of this work is that the toxicity of Ag NCs primarily originated from the nanoclusters themselves rather than from dissolved Ag^+^. The toxicity of Ag nanoparticles (Ag NPs) is generally considered to be primarily associated with the release of Ag^+^ [5,19,37]. In contrast, ICP-MS analysis demonstrated that only 0.36% of total silver was released as Ag^+^ after 72 h (Figure S7A), reflecting the high stability of the GSH-protected Ag NCs. Comparative toxicity assays further confirmed that the released Ag^+^ could not account for the severe inhibitory effects induced by Ag NCs. The trace amounts of Ag^+^ released from the Ag NCs did not exert a significant effect on the growth of Syn7942; Ag^+^ at a total silver concentration equivalent to that of the Ag NCs induced only slight growth inhibition. Moreover, this Ag^+^-induced toxicity was completely abolished by L-Cys addition. In contrast, the pronounced inhibition of growth and photosynthesis caused by Ag NCs persisted even in the presence of L-Cys (Figure 5). As a sulfur-containing amino acid, L-Cys possesses a thiol side chain with high affinity for Ag^+^, enabling it to chelate free Ag^+^ and form stable complexes, thereby reducing bioavailable Ag^+^ and mitigating ionic silver toxicity [19]. These findings indicate that the toxicity of Ag NCs is primarily attributed to their own intrinsic properties rather than stemming from the release of Ag^+^; this suggests that their mechanism of toxicity differs from that of Ag NPs, whose toxic effects are predominantly mediated by released Ag^+^. RT-qPCR analysis further revealed the molecular basis of Ag NC toxicity. In Syn7942, the PSII reaction center D1 protein is encoded by a multigene family, in which *psbA1* predominates under standard conditions and encodes the D1:1 isoform, whereas *psbA2* and *psbA3* encode the stress-responsive D1:2 isoform [38]. These three *psbA* genes are known to be differentially regulated under environmental stress; strong light and low temperature induce psbA2/3 expression while downregulating *psbA1*, representing a canonical acclimation strategy for D1 replacement [38]. Compared with Ag^+^, Ag NCs resulted in a more significant downregulation of *psbA1*, accompanied by the substantial upregulation of *psbA2* and *psbA3*. Given that Ag NCs possess semiconductor-like properties (e.g., size-dependent bandgap and generation of electron-hole pairs by light excitation), internalized Ag NCs may act as exogenous photosensitizers under illumination. They could interrupt the photosynthetic electron flow at the acceptor side of PSII, thereby mimicking a state of photoinhibition and triggering the stress-induced substitution of D1:1 with D1:2. This hypothesis is corroborated by the transcriptional upregulation of *psbA2*/*psbA3* and downregulation of *psbA1*, a pattern of transcriptional regulation typically elicited by photoinhibition [39,40]. This transcriptional change indicates that the stress-responsive D1 replacement mechanism has been activated, suggesting that Ag NCs have inflicted more severe damage upon PSII. This stress response is exacerbated by the simultaneous inhibition of other key PSII components. These co-occurring molecular perturbations align with the physiological observations, specifically, that Ag NC treatment leads to a significant decline in F_v_/F_m_ values and electron transport rates (Figure 3), indicating severe impairment of PSII function.

Beyond PSII, Ag NCs caused a widespread suppression of genes encoding key components of the cytochrome b6f complex (*petC*) and PSI subunits (*psaA*, *psaB*, *psaC*). In contrast, Ag^+^ exposure induced the upregulation of *psaA* and *petC*, which may reflect a compensatory response to maintain linear electron flow; whereas Ag NCs suppressed these genes, indicating a more severe disruption of the photosynthetic electron transport chain. Furthermore, *isiA*, which encodes a protein playing a pivotal role in light harvesting and photoprotection, was significantly downregulated under Ag NC exposure [41]. Given that *isiA* is typically induced under oxidative stress [42], its suppression suggests an impaired photoprotective capacity, rendering the photosynthetic apparatus more susceptible to damage. Increased ROS levels lead to the transcriptional upregulation of the antioxidant gene *sodB*, encoding Fe-superoxide dismutase. This response aligns with the elevated SOD activity and reflects the cells’ accelerated scavenging of superoxide radicals generated by the impaired photosynthetic electron transport chain (Figure 4C). In addition to triggering antioxidant defenses, Ag NCs also impaired the cellular capacity for metal detoxification.

The metallothionein gene *smtA* was significantly downregulated. This downregulation was even greater than that observed under Ag^+^ exposure. In Syn7942, SmtA is a well-characterized metallothionein that binds metal ions (such as Zn^2+^, Ag^+^) via cysteine- and histidine-rich coordination [43]. The *smtA* gene resides within the *smt* locus. This locus contains a divergently transcribed repressor, *smtB*. *smtB* controls *smtA* transcription in response to metal availability [44]. Therefore, downregulation of *smtA* may have impaired intracellular metal chelation capacity, thereby leading to an increased intracellular silver burden and consequently exacerbating nanocluster-specific toxicity.

The impact of Ag NCs further extended to the dark reactions of photosynthesis. Both RuBisCO subunits (*rbcL*, *rbcS*) were significantly downregulated, whereas Ag^+^ only affected *rbcL*. Within the carbon-concentrating mechanism, the carboxysome core protein (*ccmM*) is suppressed, while the shell protein (*ccmO*) is upregulated, indicating that the carboxysome assembly process has been perturbed. Collectively, these changes indicate impaired CO_2_ fixation, which limits carbon flux and promotes ROS accumulation due to a deficiency of electron acceptors.

The RT-qPCR data reveal that Ag NCs induce a unique toxic response compared to Ag^+^. By impeding photosynthetic electron transport, interfering with carbon fixation, and impairing metal detoxification, the damage inflicted by Ag NCs has exceeded the repair and adaptation capabilities of cells. This coordinated, multi-level perturbation provides the molecular basis for the photosynthesis and growth inhibition effects induced by Ag NCs. Overall, this study demonstrates that the ultrasmall Ag NCs exert a distinct nanotoxicity mechanism toward cyanobacteria.

In this study, the ecotoxicological impacts of Ag NCs on cyanobacteria were evaluated within a concentration range of 0.05 to 5 mg L^−1^. It is worth noting that the predicted environmental concentrations of silver nanomaterials in aquatic ecosystems typically range from ng L^−1^ to low µg L^−1^ levels [15,16]. However, concentrations can be significantly higher in localized pollution hotspots, near wastewater effluents, or at accidental spill sites. Therefore, the lower bound of tested concentrations (0.05 mg L^−1^) may serve as a relevant indicator of potential risks in localized hotspot environments. Although the maximum concentration (5 mg L^−1^) exceeds typical environmental concentrations, this deliberate overestimation is indispensable for toxicological evaluation. It successfully triggered and clearly elucidated the underlying toxicological mechanisms of Ag NCs in cyanobacteria. Specifically, high-concentration exposure induced excessive intracellular ROS accumulation, triggering oxidative stress that activated the antioxidant enzymatic defense system, which consequently impaired chlorophyll synthesis and photosynthetic efficiency. More importantly, from an ecological perspective, this higher exposure concentration holds profound environmental significance. As primary producers at the water ecosystems, cyanobacteria can efficiently internalize and accumulate Ag NCs. These accumulated nanomaterials possess a high potential to transfer to higher trophic levels via bioaccumulation and biomagnification along the food chain, ultimately posing a systemic threat to consumers such as zooplankton, fish, and potentially humans. Therefore, the 5 mg L^−1^ exposure setting not only provides an early-warning benchmark for localized extreme discharge events, but also establishes an essential safety margin for assessing the long-term, cumulative ecological risks of Ag NCs as they propagate through the aquatic food web.

## 5. Conclusions

This study demonstrates that the ultrasmall Ag NCs exert distinct nanotoxic effects on the model cyanobacterium Syn7942 through mechanisms different from those of dissolved Ag^+^. Benefiting from their ultrasmall size, Ag NCs can efficiently penetrate cyanobacterial cells and directly interfere with the photosynthetic machinery located on thylakoid membranes, leading to severe inhibition of photosynthetic electron transport, suppression of photosynthesis-related gene expression, and impairment of PSII repair and assembly. These disruptions further promote excessive ROS generation and oxidative stress. Although antioxidant enzyme activities were upregulated as a compensatory response, the significant downregulation of the metallothionein gene *smtA* impaired intracellular Ag detoxification, ultimately causing oxidative damage to exceed the cellular defense capacity. In addition, Ag NCs induced broader disturbances in carbon fixation and photosynthetic regulation than equivalent concentrations of Ag^+^, confirming that their toxicity primarily originates from the intrinsic physicochemical properties of the nanoclusters rather than Ag^+^ release. Given the pivotal role cyanobacteria play as producers in aquatic ecosystems, the internalization of Ag NCs into cyanobacterial cells and their subsequent transfer through the food chain may pose potential risks to the ecosystem. These findings provide mechanistic insights to a deeper understanding of the environmental impact of emerging ultrasmall nanomaterials.

## Data Availability

The original contributions presented in this study are included in the article. Further inquiries can be directed to the corresponding authors.

## Supplementary Materials

The following supporting information can be downloaded at: https://www.mdpi.com/article/10.3390/toxics14080684/s1, Figure S1: Comparison of experimental and theoretical isotope patterns for the identified Ag NCs species. Panel (A) corresponds to the [Ag_11_(SG)_6_ + 3Na−8H]^2−^ cluster, (B) displays the pattern for [Ag_11_(SG)_6_ + 2Na−7H]^2−^, (C) displays the pattern for [Ag_11_(SG)_6_ + Na−6H]^2−^, (D) displays the pattern for [Ag_11_(SG)_6_−5H]^2−^. In both panels, the experimental data is depicted by the black profile, overlaid with the red curve representing the theoretical simulation; Figure S2: Growth curve of Syn7942 cells exposed to varying concentrations of Ag NCs; Figure S3: Chl *a* content of Syn7942 cells exposed to varying concentrations of Ag NCs; Figure S4: Growth inhibition rates of Syn7942 after 72 h of exposure to Ag NCs; Figure S5: Dark respiratory oxygen consumption rate; Figure S6: Effective quantum yield of PSII [Y(II)] and Effective quantum yield of PSI [Y(I)]; Figure S7: A. Concentration of Ag^+^ released from 4 mg L^−1^ Ag NCs. B. Total Ag content in the 4 mg L^−1^ Ag NCs; Figure S8: OD_730_ of Syn7942 exposed to four conditions: BG11 control, simulated Ag^+^ release (0.2 μg L^−1^ Ag^+^), total equivalent silver content (55 μg L^−1^ Ag^+^), and 4 mg L^−1^ Ag NCs, both in the presence and absence of L-Cys as a thiol chelator. Table S1: The equation of F_v_/F_m_, Y(II), Y(I); Table S2. Primer sequences used for RT-qPCR analysis.

## Abbreviations

The following abbreviations are used in this manuscript:

Ag NCs	Silver nanoclusters
Syn7942	*Synechococcus elongatus* PCC 7942
Ag^+^	Silver ions
Ag NPs	Silver nanoparticles
PSII	Photosystem II
PSI	Photosystem I
ROS	Reactive oxygen species
AgNO_3_	Silver nitrate
GSH	Glutathione
TEM	Transmission electron microscopy
ESI-MS	Electrospray ionization mass spectrometry
OD_730_	Optical density at 730 nm
Chl *a*	Chlorophyll *a*
EC_50_	Half-maximal effective concentration
F_0_	Minimum fluorescence
F	Steady-state fluorescence
F_m_′	Maximum fluorescence under light adaptation
P_m_	Maximum P700 oxidation level
P_m_′	Maximum P700 oxidation level under light adaptation
P700_ox_	P700 oxidation level
F_m_	Maximum fluorescence
DCMU	3-(3,4-dichlorophenyl)-1,1-dimethylurea
F_v_/F_m_	Maximum quantum yield
Y(II)	Actual photosynthetic efficiency of PSII
Y(I)	Actual photosynthetic efficiency of PSI
DCFH-DA	2′,7′-dichlorofluorescein diacetate
SOD	Superoxide dismutase
MDA	Malondialdehyde
POD	Peroxidase
CAT	Catalase
BSA	Bovine serum albumin
ICP-MS	Inductively coupled plasma mass spectrometry
STEM	Scanning transmission electron microscopy
L-Cys	L-cysteine
RT-qPCR	Reverse transcription quantitative real-time PCR
ETR(II)	Electron transport rate of PSII
ETR(I)	Electron transport rate of PSI
CCM	Carbon-concentrating mechanism
Cytb6f	Cytochrome b6f
