# Supplementary material for: Nanotoxicity of Ultrasmall Silver Nanoclusters to Cyanobacteria
Source: Toxics. 2026 Aug 3;14(8):684. doi: 10.3390/toxics14080684 (PMC13517853; doi:10.3390/toxics14080684)
Supplement: Supplementary file 1 [file toxics-14-00684-s001.zip › toxics-4396809-supplementary.pdf]

Nanotoxicity of Ultrasmall Silver Nanoclusters to Cyanobacteria

Xiaofei Wu [1,2], Lili Zhang [2,3,4], Mengqi Yin [2,5], Xiaojuan Han [1,2,3], Guojie Zhou [2], Guodong Luan [2,3,4], Xiaoyan Sun [2,3,4], Weihua Wang [2,3,4,*], Zuozhen Han [1,6,*] and Xuefeng Lu [2,3,4,6]

1 College of Earth Science and Engineering, Shandong University of Science and Technology, Qingdao 266590, China; wuxf@qibebt.ac.cn (X.W.); hanxj@qibebt.ac.cn (X.H.)
2 State Key Laboratory of Photoelectric Conversion and Utilization of Solar Energy, Qingdao Institute of Bioenergy and Bioprocess Technology, Chinese Academy of Sciences, Qingdao 266101, China; zhangll2@qibebt.ac.cn (L.Z.); yinmq@qibebt.ac.cn (M.Y.); 15663036171@163.com (G.Z.); luangd@qibebt.ac.cn (G.L.); sunxy@qibebt.ac.cn (X.S.); lvxf@qibebt.ac.cn (X.L.)
3 Shandong Energy Institute, Qingdao 266101, China
4 Qingdao New Energy Shandong Laboratory, Qingdao 266101, China
5 School of Biological Science and Technology, University of Jinan, Jinan 250022, China
6 Qingdao Marine Science and Technology Center, Qingdao 266237, China
* Correspondence: wangwh@qibebt.ac.cn (W.W.); hanzuozhen@sdust.edu.cn (Z.H.)

Supplementary Figures.

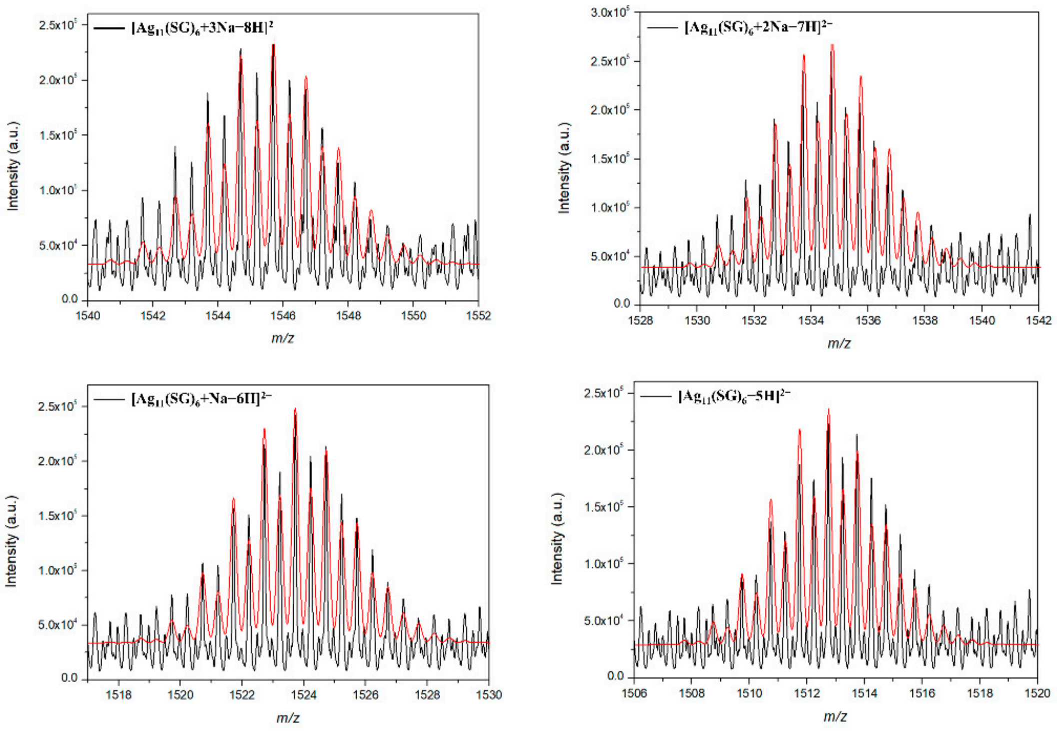

Figure S1. Comparison of experimental and theoretical isotope patterns for the identified Ag NCs species. Panel (A) corresponds to the $[Ag_{11}(SG)_6+3Na-8H]^{2-}$ cluster, (B) displays the pattern for $[Ag_{11}(SG)_6+2Na-7H]^{2-}$, (C) displays the pattern for $[Ag_{11}(SG)_6+Na-6H]^{2-}$, (D) displays the pattern for $[Ag_{11}(SG)_6-5H]^{2-}$. In both panels, the experimental data is depicted by

the black profile, overlaid with the red curve representing the theoretical simulation.

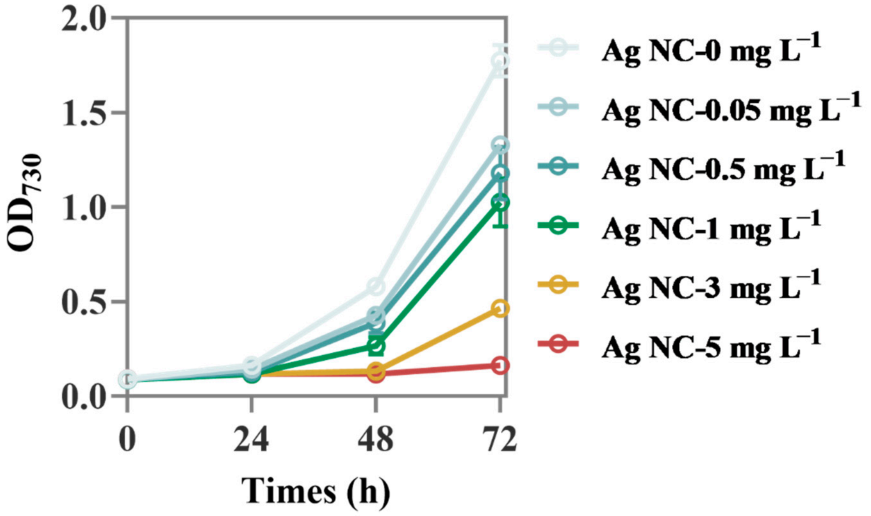

Figure S2. Growth curve of Syn7942 cells exposed to varying concentrations of Ag NCs.

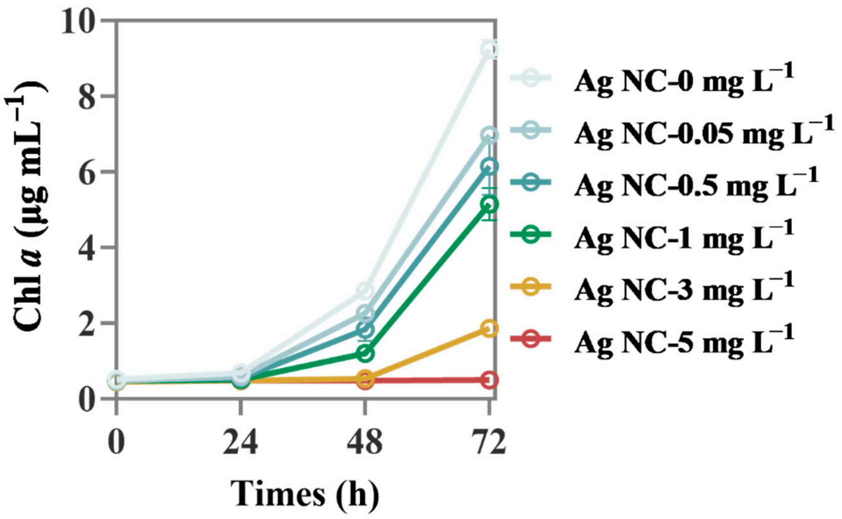

Figure S3. Chl *a* content of Syn7942 cells exposed to varying concentrations of Ag NCs.

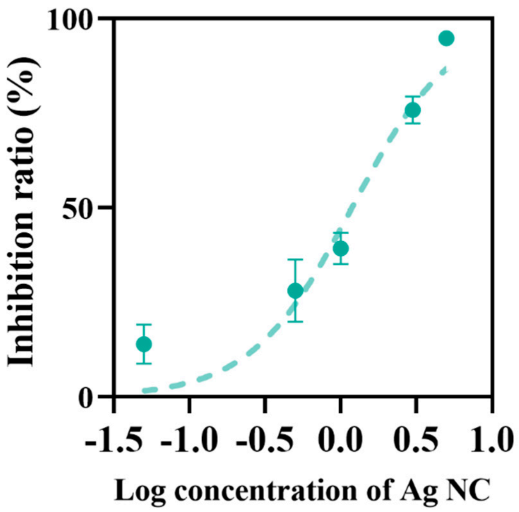

Figure S4. Growth inhibition rates of Syn7942 after 72 h of exposure to Ag NCs.

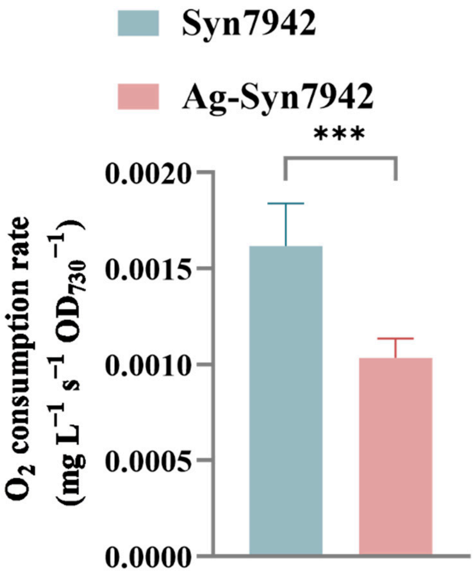

Figure S5. Dark respiratory oxygen consumption rate.

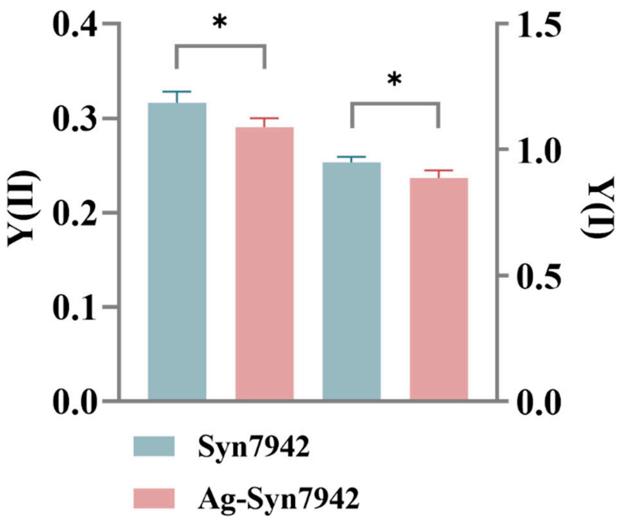

Figure S6. Effective quantum yield of PSII [Y(II)] and Effective quantum yield of PSI [Y(I)].

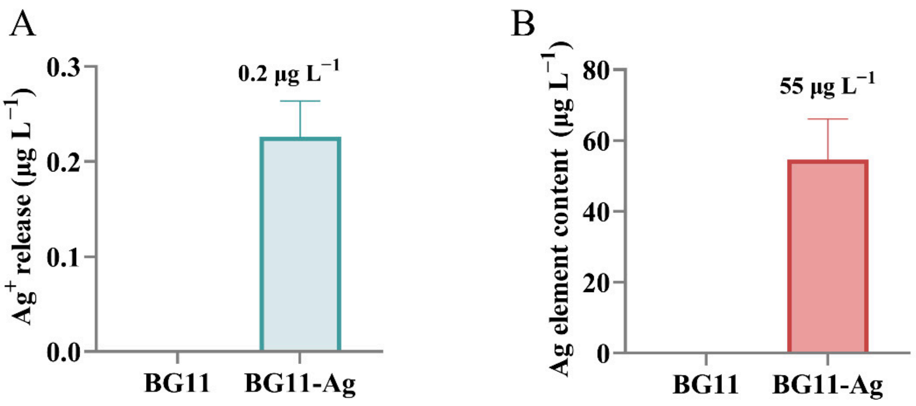

Figure S7. A. Concentration of Ag^+ released from 4 mg L^{-1} Ag NCs. B. Total Ag content in the 4 mg L^{-1} Ag NCs.

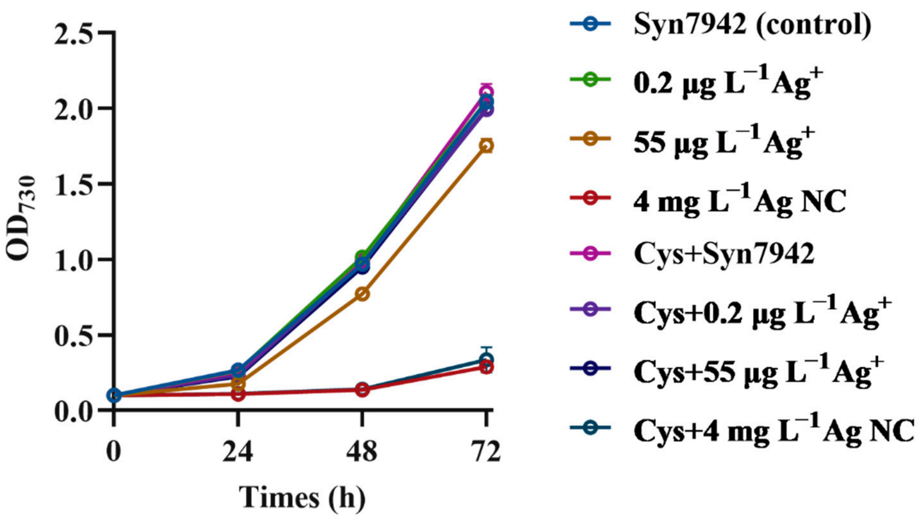

Figure S8. OD_{730} of Syn7942 exposed to four conditions: BG11 control, simulated Ag^+ release (0.2 μg L^{-1} Ag^+), total equivalent silver content (55 μg L^{-1} Ag^+), and 4 mg L^{-1} Ag NCs, both in the presence and absence of L-Cys as a thiol chelator.

Supplementary Table.

Table S1. The equation of F_v/F_m, Y(II), Y(I).

Parameter	Equation
F_v/F_m	$(F_m-F_0)/F_m$
Y(II)	$(F_m'-F)/F_m'$
Y(I)	$1-P_m/P700_{ox}-P_m/P_m'$

Table S2. Primer sequences used for RT-qPCR analysis.

gene	Gene function	5′ → 3′ Sequence (Forward/Reverse)
SecA	Internal control	ACGACGGTCAGATTGCCGAGAT/GCGACATTCCCTGCTGGATTAG
PsbA1 (*Synpcc7942_0424*)	PSII D1 protein (isoform 1)	TCTATCCGATTTGGGAAGCCGCTA/CCCATTGACGACCCATGTAGCAG
PsbA3 (*Synpcc7942_0893*)	PSII D1 protein (isoform 3, stress-responsive)	CTTCCAGCAACGCCATTGGT/CCATGTAGCAGAAAACGCCGAT
PsbA2 (*Synpcc7942_1389*)	PSII D1 protein (isoform 2, stress-responsive)	ATCATCTCTGGTGCTGTTGTTCC/ACCGTTGTACAGCCACTCGTC
PsbD1 (*Synpcc7942_0655*)	PSII D2 protein	CAATCGAACACCTTCCGGGCAT/TCACCGGCACGAACAGCAT
PsbB (*Synpcc7942_0697*)	PSII CP47 antenna protein	CCATATCTGGCACGGCTCT/TAAGCGGCTGTTTTCCGAGT
PetC (*Synpcc7942_1232*)	Cytochrome b6f complex (Rieske iron-sulfur protein)	CTTGGCAATGATGTCGTCCT/CGCTTCGCTACTTTCGACCAC
PsaA (*Synpcc7942_2049*)	PSI reaction center subunit	ATGACGATAAGCCCACCGGAG/GACCCGGCTTACCCCACT
PsaB (*Synpcc7942_2048*)	PSI reaction center subunit	CTTCTTGATCCGCGACTATGACC/ATGATCGCTTCTTTGTGCTCC
PsaC (*Synpcc7942_0535*)	PSI iron-sulfur center subunit	TGCCCTCTCGATGTACTGGAA/CCAGGTAGACCCGAATGCTCA
IsiA (*Synpcc7942_1542*)	Iron-stress-induced protein (photoprotection)	CATTTGGCACATTTTGGTTCCG/GCCACCCAAGGAGTACGACA
SmtA (*Synpcc7942_1290*)	Metallothionein (metal detoxification)	GTGAGCCCTGTCTCTGCAACG/CCGCAGCCTTTGCTACCACCG
sodB (*Synpcc7942_0801*)	Fe-superoxide dismutase (antioxidant)	TGTTCAACAATGCGGCTCA/CTTGTTTGAACTCGGTCACGAA
rbcL (*Synpcc7942_1426*)	RuBisCO large subunit (carbon fixation)	GATGCAATCCACAAATCGCAAG/AGCCGTCAAGAAGTCATGC

rbcS (*Synpcc7942_1427*)	RuBisCO small subunit (carbon fixation)	CTTCCACCCCTTGATCGAGT/ATGTAGCAATCACCGTATTCGC
ccmM (*Synpcc7942_1423*)	Carboxysome core protein (carbon-concentrating mechanism)	ACCTCCAGTGATTGTCCGGTCT/CTCACGGTCGTTGCCTCGCTA
ccmO (*Synpcc7942_1425*)	Carboxysome shell protein (carbon-concentrating mechanism)	CCATGCTCAAATCGGCTAACGTC/CACATTAGAAACCGTGCCTTG
